# Laparoscopic Surgery for Ovarian Pregnancy using Diathermy Hook with Conservation of Ovary: A Case Report and Literature Review

**DOI:** 10.3390/jcm2040214

**Published:** 2013-10-31

**Authors:** Alpha K. Gebeh, Akwasi A. Amoako, Olakanmi Joseph, Asok Banerjee

**Affiliations:** 1 Division of Women’s and Perinatal Services, Department of Obstetrics & Gynecology, University Hospitals of Leicester NHS Trust, Leicester Royal Infirmary, Infirmary Square, Leicester, LE1 5WW, UK; E-Mails: alphagebeh@gmail.com (A.K.G.); olakanmi@aol.com (O.J.); 2 Leeds Centre for Reproductive Medicine, Leeds Teaching Hospitals NHS Trust, Seacroft Hospital, York Road, Leeds, LS14 6UH, UK; E-Mail: akwasi.amoako12@gmail.com

**Keywords:** ovarian pregnancy, ectopic pregnancy, diathermy hook, laparoscopic surgery

## Abstract

A 31-year-old woman presented with a 7-week history of irregular vaginal bleeding without abdominal pain. She had been using the intrauterine contraceptive device (IUD) for the last 3 years. A pregnancy test was positive and subsequent serum beta human chorionic gonadotropin (β-HCG) was 4992 mIU/mL. A transvaginal ultrasound scan demonstrated an empty uterus with an associated adnexal mass but no free fluid. A right primary ovarian ectopic pregnancy was diagnosed a laparoscopy. This was managed laparoscopically using monopolar diathermy hook with conservation of the ovary and minimal blood loss. Ovarian pregnancy is rare, especially in women without the classical risk factors for tubal pregnancy, and efforts should be made to exclude ectopic pregnancy in the absence of abdominal pain in a woman of reproductive age presenting with prolonged and irregular vaginal bleeding. Methods to conserve the ovary are also encouraged in cases of ovarian pregnancy.

## 1. Introduction

Ovarian pregnancy is rare and remains a diagnostic and management challenge, especially in settings where health resources are stretched [1]. A potential major surgical and clinical problem is intra-peritoneal bleeding and difficulty in achieving haemostasis to conserve the ovary. This is in addition to the difficulty in accurately arriving at a diagnosis pre-operatively. The aim of this article is to describe a case of an unruptured primary ovarian pregnancy managed laparoscopically using diathermy hook with conservation of the ovary. In addition, it would add to the literature of methods to conserve the ovary in cases of ovarian pregnancy.

## 2. Case

A 31-year-old African woman presented with a seven-week history of irregular vaginal bleeding without abdominal pain. She was married and gave vaginal birth twice. The patient had been using a copper intrauterine contraceptive device (IUD) for contraception and her medical history was unremarkable. 

**Figure 1:**
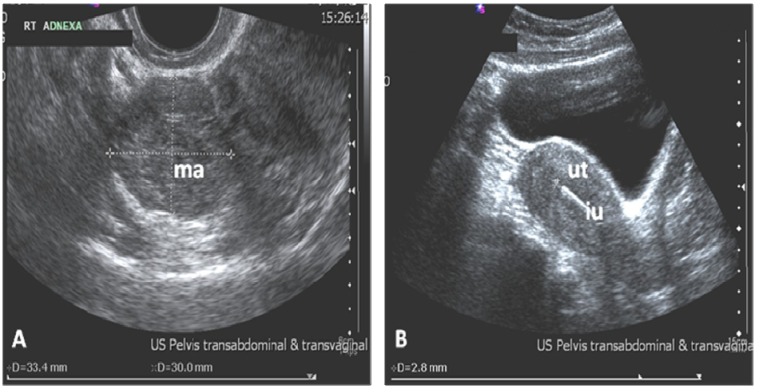
Ultrasound image demonstrating a mass (ma) in the right adnexa (**A**); (**B**) shows a thin endometrium with a normally sited intrauterine contraceptive device (IUD) (iu) in the uterus (ut).

On examination, the patient was haemodynamically stable. The abdomen was soft and non-tender and pelvic examination was unremarkable. A urine pregnancy test was positive with an initial β-HCG of 4992 mIU/mL. A subsequent trans-vaginal ultrasound scan demonstrated normal size uterus with the IUD in the correct position, an endometrium measuring 2.8 mm with no intra-uterine gestation sac. The left ovary appeared normal and a 33.4 mm × 30 mm × 28 mm mass adjacent to the right ovary was noted with no free fluid in the pelvis (Figure 1). The (copper T 380) IUD had been removed and a repeat β-HCG in 48 h was 4232 mIU/mL. 

**Figure 2:**
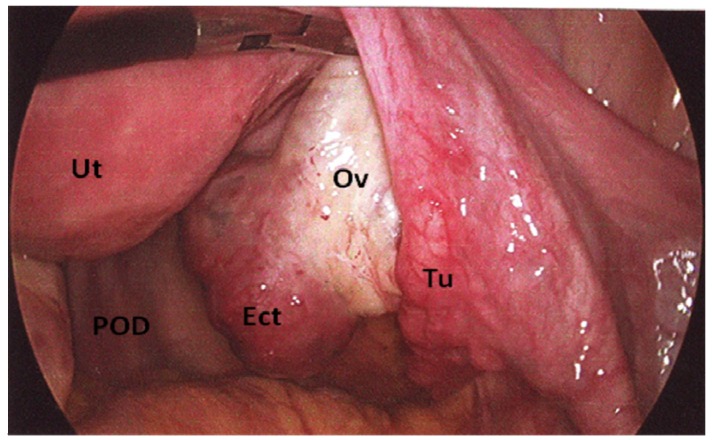
Laparoscopic view of an unruptured right ovarian ectopic pregnancy. (Ut = uterus, POD = pouch of Douglas, Ect = ectopic pregnancy, Ov = right ovary, Tu = Fallopian tube).

Considering a slow fall of HCG, a diagnostic laparoscopy was planned. Laparoscopy was performed through a 10 mm trans-umbilical port and two further secondary (right-5 mm, left-10 mm adjustable) ports inserted on each iliac fossa, lateral to epigastric vessels. The uterus and both the Fallopian tubes appeared normal without dilatation or signs of intra-tubal hemorrhage and no bleeding from the fimbriae. A haemorrhagic mass (20 mm × 30 mm) of dark red color with intact surface attached to the right ovary was noted (Figure 2). The left ovary clinically appeared normal. The pouch of Douglas contained 30 to 40 mL of serous peritoneal fluid and no evidence of endometriosis or pelvic adhesions was noted. A clinical diagnosis of right ovarian pregnancy was made. 

The ovary was stabilized by holding the ovarian ligament with atraumatic grasping forceps. The suspected ovarian pregnancy was dissected off intact from the right ovarian surface, by monopolar diathermy hook with conservation of the ovary. Normal saline irrigation was carried out and ovarian haemostasis checked. There was hardly any intra-operative bleeding noted from the ovary. The specimen retrieved through 10 mm port by means of an endopouch^®^ (Ethicon Endo-Surgery, Norderstedt, Germany). On postoperative day one β-HCG dropped down to 1547 mIU/mL. Histology demonstrated the presence of chorionic villi and ovarian stromal tissue including part of the corpus luteum confirming ovarian ectopic pregnancy. She was followed up for 3 weeks until serum HCG was below 5 mIU/mL.

## 3. Discussions

Primary ovarian pregnancy is when the primary nidation occurs in the ovary as opposed to secondary ovarian pregnancy where the initial implantation is in the Fallopian tube and the ovarian attachment is secondary to tubal abortion. Ovarian pregnancies can be associated with IUD *in situ* (4%–20%), but there is however no direct evidence linking IUD with ovarian pregnancy [2]. Cases of ovarian pregnancies have also been described following ovarian hyperstimulation with gonadotropins or clomiphene citrate treatment and after assisted conception procedures [3,4,5]. The clinical manifestations may be similar to those expected in tubal pregnancy, *i.e.*, abdominal pain and vaginal bleeding, but in this case, the patient conceived with an IUD *in situ* and presented only with prolonged painless vaginal bleeding. The rarity of this condition, the variability in clinical presentation and the lack of a discriminatory test make the condition more difficult to diagnose pre-operatively [1,3,6]. Moreover, although β-HCG remains a useful adjunct for diagnosis of ectopic pregnancy, there are reports of ruptured ovarian pregnancies even with decreasing β-HCG levels [7]. This clearly adds to the diagnostic dilemma.

Most of the clinical diagnosis of an ovarian pregnancy is made during surgery when a haemorrhagic mass is noticed attached to one of the ovaries in the presence of normal looking fallopian tubes. It could be difficult to distinguish an ovarian pregnancy from a haemorrhagic corpus luteum or ovarian cyst [1,3,8]. Since the first publication of laparoscopic surgery for early ovarian pregnancy in 1988 [9], laparoscopic therapy is now the method of choice after diagnosis [10,11]. Laparoscopic surgery includes partial ovariectomy (wedge resection), ovarian cystectomy or blunt dissection of the trophoblastic tissue. Ovariectomy or adnexectomy is considered for advanced or ruptured cases [1,3,6,9]. The main problem during surgery is to procure ovarian haemostasis; which is usually achieved by applying haemostatic suture laparoscopically or using diathermy forceps (bi-polar or mono-polar) [1,3,6]. In general, diathermy or electrosurgery is the use of high frequency electric current to produce heat. These frequencies are usually greater than 100 kHz in order to avoid spasm or paralysis of muscle. With bipolar diathermy, current passes between the tips of the instruments, e.g., forceps unlike monopolar where electricity exits the patient via the indifferent electrode placed on the patient. The main clinical use of diathermy is to cut or destroy tissue to produce coagulation but the actual or specific effects, *i.e.*, coagulation or cutting effect will depend on the current intensity and wave form used. In the case series by Seinera *et al.* [10], removal of the products using biopsy forceps was the mainstay of treatment at laparoscopy in the majority of cases with haemostasis achieved using thermal coagulation. There were no cases reported of persistent trophoblast [10]. The good outcome from this case series is reassuring as it offers women the opportunity to preserve their fertility. Similarly, in Odejnimi *et al.*’s [11] case series, 11 out of 12 cases of ovarian pregnancy were successfully managed laparoscopically with conservation of the ovary while one had oophorectomy [11]. In this case, we used diathermy hook (mono-polar) to resect the ovarian pregnancy intact providing diathermy and surgical resection with minimal bleeding and conservation of ovary. Most case reports do not specifically describe the type of surgical instrument used and therefore hard to assess the types of instruments used for conservative management and how they differed if any from ours. However, this is not unexpected because the case reports or series generally focuses on the general management of ovarian pregnancy and their associated outcomes rather than the specific surgical tools or forceps used for their treatment. Whether the type of instrument used is clinically relevant or provides better surgical haemostasis is open to further debate. Certainly, the publication of more case series may provide more insight into ovarian pregnancy management and the associated surgical techniques employed.

## 4. Conclusions

Primary ovarian pregnancy is very rare, difficult to diagnose and associated with intraoperative complications including bleeding, risks of losing the ovary. In this case, we used diathermy hook (mono-polar) to dissect the ovarian pregnancy intact with conservation of ovary; a technique that is easy to learn by the trainee as well. This case adds to the literature of reports describing methods to conserve the ovary in cases of ovarian pregnancy. More importantly, it also highlights the importance of having an index of suspicion for ectopic pregnancy in women of reproductive age presenting with vaginal bleeding even in the absence of abdominal pain. This is more so the case when other risk factors for ectopic pregnancy, e.g., IUD, are present.
